# Development of a novel rodent rapid serial visual presentation task reveals dissociable effects of stimulant versus nonstimulant treatments on attentional processes

**DOI:** 10.3758/s13415-023-01152-x

**Published:** 2024-01-22

**Authors:** Abigail Benn, Emma S. J. Robinson

**Affiliations:** University of Bristol, School of Physiology, Pharmacology and Neuroscience, Biomedical Sciences Building, University Walk, Bristol, BS8 1TD UK

**Keywords:** Rapid serial visual presentation, Attention, Amphetamine, Atomoxetine, Continuous performance tasks

## Abstract

The rapid serial visual presentation (RSVP) task and continuous performance tasks (CPT) are used to assess attentional impairments in patients with psychiatric and neurological conditions. This study developed a novel touchscreen task for rats based on the structure of a human RSVP task and used pharmacological manipulations to investigate their effects on different performance measures. Normal animals were trained to respond to a target image and withhold responding to distractor images presented within a continuous sequence. In a second version of the task, a false-alarm image was included, so performance could be assessed relative to two types of nontarget distractors. The effects of acute administration of stimulant and nonstimulant treatments for ADHD (amphetamine and atomoxetine) were tested in both tasks. Methylphenidate, ketamine, and nicotine were tested in the first task only. Amphetamine made animals more impulsive and decreased overall accuracy but increased accuracy when the target was presented early in the image sequence. Atomoxetine improved accuracy overall with a specific reduction in false-alarm responses and a shift in the attentional curve reflecting improved accuracy for targets later in the image sequence. However, atomoxetine also slowed responding and increased omissions. Ketamine, nicotine, and methylphenidate had no specific effects at the doses tested. These results suggest that stimulant versus nonstimulant treatments have different effects on attention and impulsive behaviour in this rat version of an RSVP task. These results also suggest that RSVP-like tasks have the potential to be used to study attention in rodents.

**Supplementary Information:**

The online version contains supplementary material available at 10.3758/s13415-023-01152-x.

## Introduction

Attentional impairments are observed across a wide range of psychiatric and neurological disorders. The rapid serial visual presentation task (RSVP) and continuous performance tasks (CPT) have been used to study neuromodulatory systems implicated in attention (Barnes et al., 2012; Bekker et al., 2005; Boucart et al., 2000) or disorders involving perturbed attentional processing (Jimenez et al., 2016; Kahn et al., 2012; Peters et al., 2012; White & Levin 1999). In these tasks, inattention reflects a failure to respond to a target stimulus (errors of omission) and inhibit responding to nontargets (errors of commission) (Jimenez et al., 2016; Young, Geyer et al., 2013). Drugs modulating attention have been shown to affect one or both of these performance measures (Rapoport et al., 1980; Tomlinson et al., 2014; Young, Meves et al., 2013).

There have been a number of different attentional tasks developed for rodents (Demeter et al., 2008; Kim et al., 2015; Mar et al., 2017; Young, Geyer et al., 2013). One of the most widely used is the 5-choice serial reaction time task (5-CSRTT), which measures visuospatial attention in rodents, requiring them to attend to a light cue presented in one of five spaced apertures (Cole & Robbins, 1987). However, several challenges exist with these tasks, including the use of discrete trials, fixed intervals, and extensive training methods. These can lead to animals using procedural learning and developing timing strategies (Cope et al., 2016), although the use of a variable intertrial interval can help to mitigate issues with timing strategies (Benn & Robinson, 2014; Robinson, 2012). In order to look at ways to increase attentional load and reduce animals reliance on a timing strategy, a 5-choice continuous performance task (5-CCPT) has been developed which includes no-go (nontarget) trials to align more with response indices in human tasks, e.g., Connor’s CPT (Hayward et al., 2017; Young, Geyer et al., 2013; Young, Meves et al., 2013). There also has been rodent touchscreen CPT (rCPT) developed, which consists of presenting several images (target or distractor images) across a single trial but with an ITI between image presentations (Mar et al., 2017) or a target image with flanking distractors (Kim et al., 2015). Performance parameters, such as false-hit and correct-rejections, are used to infer discrimination sensitivity (d’) from Signal Detection Theory, made possible by presentation of discrete go (target) and no-go (distractor) trials (Bhakta & Young, 2017; Mar et al., 2017; Nestor et al., 1990; Young et al., 2009). Similar to a concept of a touchscreen CPT, we hypothesized that training animals in a task using a randomized image sequence (RSVP stream), containing a target and multiple distractor images, would result in a challenging and potentially translational rodent equivalent of this human attentional task. In our task, we chose stimuli presentation that resembles the single-target human RSVP attention task, where participants respond to an unpredictable target image embedded within distractor sequences. For origins of the task, see Eriksen & Collins (1969), Ostry et al. (1976), and Sperling et al. (1971). Hit rate (accuracy) and false-alarm rate are used to quantify the subject’s target detection performance within the RSVP stream (Files & Marathe, 2016). The position of the target image is temporally distributed within the image sequence and pseudo-random in relation to the positions of the distractor images (Jimenez et al., 2016).

We designed a touchscreen-based rodent task by using a continuous sequence of images where rats were trained to recognize a single image as the target whilst withholding from responding to the other images. Our prediction was that this task would be less reliant on timing strategies, require animals to sustain attention for a longer period of time, and might enable us to dissociate between mechanisms involved in enabling animals to sustained attention for the duration of the sequence (attention curves plotting accuracy against time), discriminative or perceptual accuracy (target vs. false-alarm image), and impulsive responding (where responses increase irrespective of target category). To test this, we first trained animals by using a random sequence of images containing a single target and five nontarget images. Based on these initial findings, we added one distractor with an image that contained many of the same features as the target to provide a false-alarm image. We hypothesized that this would give a clearer distinction between target (accuracy), false-alarm, and distractor responses, and the ability to distinguish inattention (responses to false-alarm image) versus impulsive responding (responses to all distractor images). We tested different pharmacological treatments that have been studied in similar rodent attention tasks and with relevance to human ADHD (Bekker et al., 2005; Bizarro et al., 2004; Ernst et al., 1994; Newcorn et al., 2008) and to compare the effects of stimulant versus nonstimulant ADHD medications. In should be noted that a limitation of the current study was that only atomoxetine and amphetamine were tested in both cohorts. The design of the task also offers both advantages and disadvantages relative to other rodent attention tasks and is not meant to be a replacement but rather add an additional option for study different aspects of attentional processing in rodent models. In this initial study, only male rats of a single strain were tested and further studies in different strains, sexes, and also mice are necessary to establish greater validation.

## Methods

### Subjects

Two cohorts of male Lister hooded rats (*n* = 12 per group) weighing approximately 300 g at the start of training (Harlan, UK) were used. Sample size was based on detecting a large effect size and data from previous studies by using the 5-CSRTT. Rats were pair housed with standard environmental enrichment (sawdust bedding, nesting material, cardboard tubes) under temperature-controlled conditions and 12-hr reverse light-dark cycle (lights off at 0700 hr). Rats were food restricted to approximately 90% of their free feeding weight matched to a normal growth curve and then maintained at a healthy weight using body condition scores (~18 g/day laboratory chow, Purina, UK) with water provided ad libitum. All animals were habituated to handling by using positive reinforcement before starting procedures. All procedures were conducted and are reported in accordance with the ARRIVE guidelines and requirements of the UK Animals (Scientific Procedures) Act 1986 and approved by the University of Bristol Animal Welfare and Ethical Review Board. Behavioral testing was performed between 8 am and 5 pm during the animals’ active phase.

### Behavioral training

**Fig. 1:**
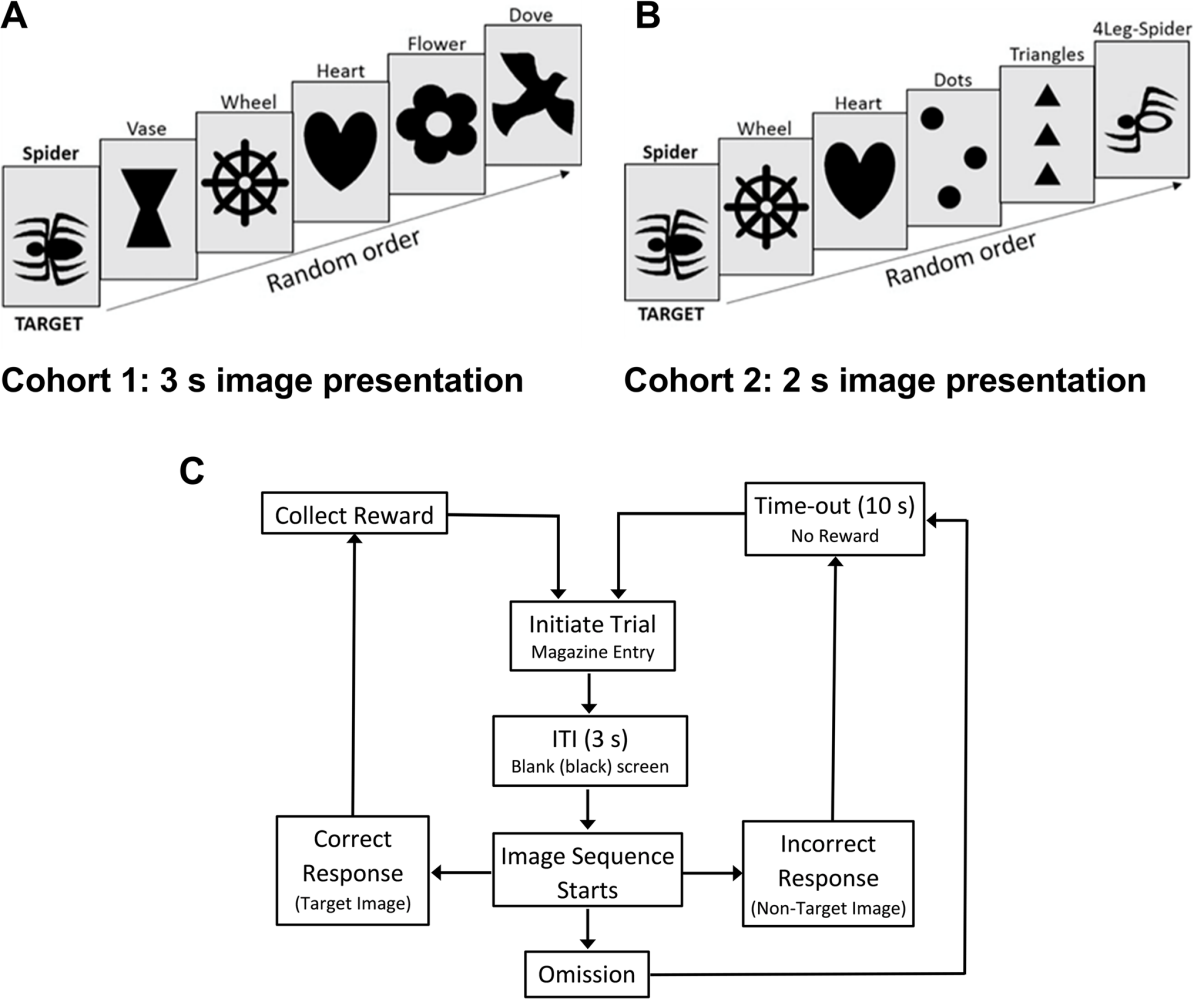
Images and trial outcomes for the rat-rapid serial visual presentation task (R-RSVP). Images used for cohort 1 (**A**) and cohort 2 (**B**) with presentation time of 3 s and 2 s, respectively. Cohort 2 images contained a false-alarm (4-leg spider) image. Flow chart representing all possible trial outcomes during task performance (**C**)

**Fig. 2:**
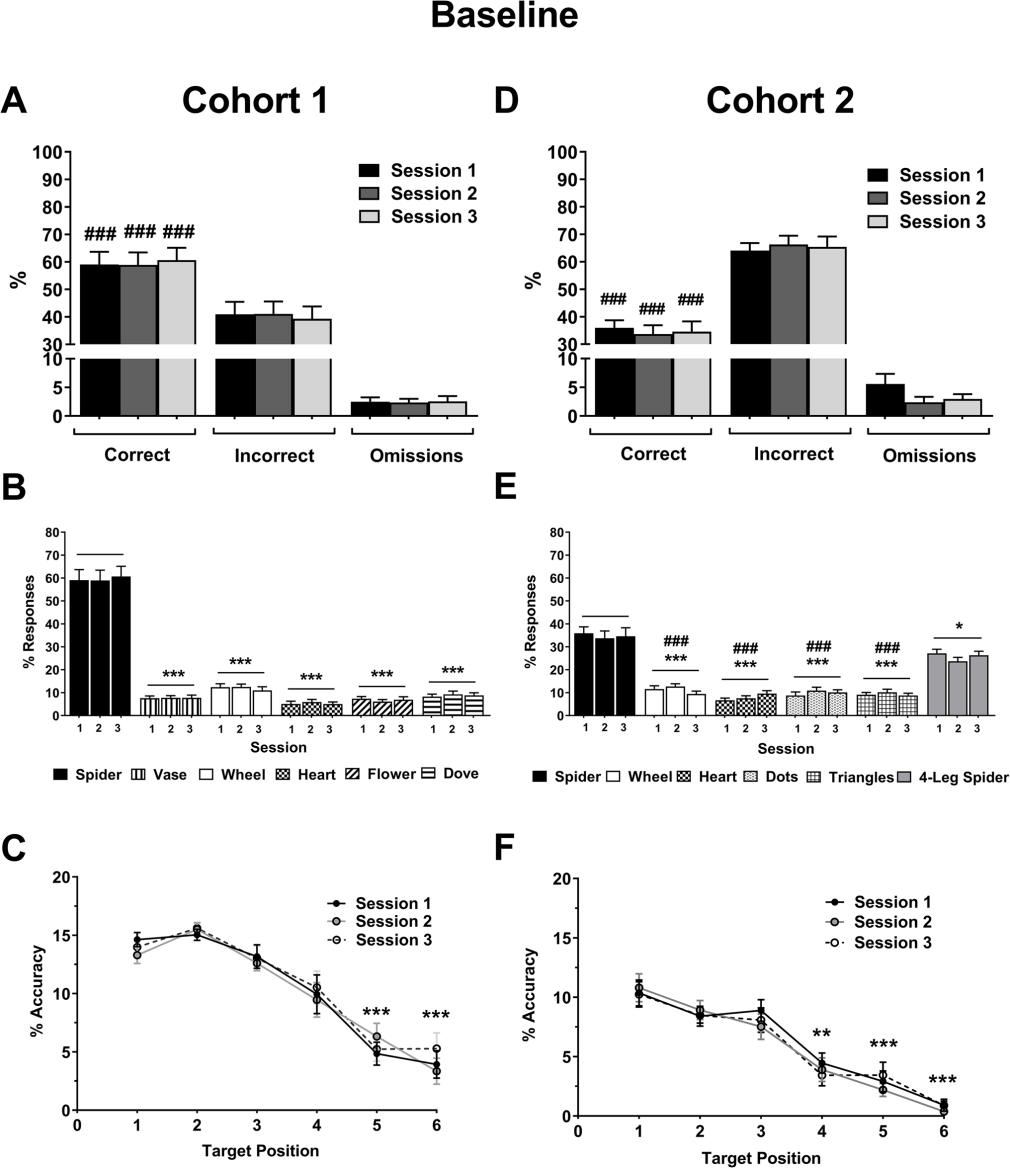
Performance data for cohort 1 (**A–C**) and cohort 2 (**D–F**) for the last three consecutive pretreatment baseline sessions. Response data for % correct, % incorrect, and % omissions for cohort 1 (**A**) and cohort 2 (**D**). Image responses for cohort 1 (**B**) and cohort 2 (**E**), spider is the target image (accuracy). The sum of distractor responses (all images except spider) is equivalent to incorrect responses in (**A**). Attention curves showing accuracy per target sequence position for cohort 1 (**C**) and cohort 2 (**F**). Results are shown for the total population, mean ± SEM, n = 12 animals per cohort, *p < 0.05, **p < 0.01, ***p < 0.001, versus target image (spider) or target sequence position (within-subject), ###*p* < 0.001 versus 4-leg spider (false alarm) or chance performance (17%, 1-sample *t*-test)

A rat-rapid serial visual presentation task (R-RSVP) was designed based on the human RSVP task (Echiverri-Cohen et al., 2016) and used a single target (Jimenez et al., 2016) and therefore differs from the attentional blink paradigm (Boucart et al., 2000; Myers et al., 2013). Touchscreen boxes (Med Associates, USA) containing three screen panels (left, right, center), controlled by KLimbic Software (Conclusive Solutions Ltd, UK), were used for all training and testing. The behavioural equipment and software were supplied by OCB Solutions Ltd, European distributors for Med Associates UK (https://www.med-associates.com/contact/). Rats were trained to screen press in response to a specific stimulus image (“spider”) embedded in a sequence of distractor images (Fig. 1). The training schedule used for both cohorts is described in detail under supplementary methods and summarized in Supplementary Table S1. All rats from cohorts 1 and 2 were successfully trained by using this graduated training procedure over a period of 42–60 sessions. Rats were trained by using images (jpeg 260x380 pixels) illustrated in Fig. 1A (cohort 1) and Fig. 1B (cohort 2). Each cohort responded to the same target image (“spider”). The introduction of an image that more closely resembled the target image versus the other distractor images was implemented to act as a false alarm (Video 1). This was designed to resemble instances where certain targets and distractors are closely related, for example, letter “S” and numeral “5” (Nakatani et al., 2012) making accuracy identification of the target harder. All animals were trained by using the same image set and target versus nontarget images were not counterbalanced. There is the potential for perceptual differences to impact on the performance and the ease with which animals differentiate between images. This can be seen with cohort 1 where the distractor images are not achieving the same level of performance (Fig. 2). For cohort 2, we changed some of the images and also added a false alarm. This achieved a baseline performance more in line with what we would predict based on performance in humans. Because we have used six different images and the false-alarm image is paired with the specific target, to fully counterbalance design would require a very large number of combinations. The design used may introduce a bias related to the specific choice of target and nontarget images; however, we use a within-subject design to look at drug effects, which will help to reduce potential confounds that this could introduce, although not completely mitigate these. Future studies could look in more detail at the images being used and optimize for perceptual similarity and a design that is more readily counterbalanced.

### Drugs

Cohort 1 received treatments in the following order: amphetamine, atomoxetine, methylphenidate, ketamine, nicotine. Cohort 2 received treatments in the following order: amphetamine, atomoxetine. Atomoxetine hydrochloride (0.3–3.0mg/kg, t = −40 min), ketamine hydrochloride (1.0–10.0 mg/kg, t = −5 min), and nicotine (0.01–0.1 mg/kg, t = −10 min) were purchased from Tocris Bioscience, UK, dissolved in 0.9% saline, and administered by intraperitoneal injection. Methylphenidate (1.0–10.0 mg/kg, t = −30 min) and amphetamine (0.3–1.0 mg/kg, t = −30 min) were purchased from Sigma-Aldrich, UK, dissolved in distilled water, and mixed with strawberry milkshake (50:50, Yazoo, Campina, UK) for oral administration. Before oral dosing using palatable solutions, animals were pretrained to drink from a syringe using the same vehicle. All drugs were prepared fresh each day and dosed in a final volume of 1 ml/kg. Doses used were based on previous behavioral studies (Benn & Robinson, 2017; Murphy et al., 2008; Robinson, 2012; Robinson et al., 2008) and administered by using a refined injection method (Stuart & Robinson, 2015). The choice of doses and route of administration were based on previous studies in the 5CSRTT and evidence that the neurochemical effects of the psychostimulants can be influenced by the route of administration. Specifically, Berridge et al. (2006) found that oral administration of methylphenidate resulted in less effects on subcortical dopamine and a relatively selective increase in noradrenaline and dopamine in the prefrontal regions (Berridge et al., 2006). Whilst the other drugs tested have most commonly been administered by the intraperitoneal route, this does not preclude a possible effect of route of administration for any of the treatments and may lead to some differences in effects in the task that have yet to be explored.

### Testing procedure

Cohort 1 animals were trained by using images shown in Fig. 1A (3-s image presentation) and performed the following dose-response experiments: amphetamine, atomoxetine, ketamine, nicotine, and methylphenidate. Cohort 2 were then trained by using images in Fig. 1B (2-s image presentation) and performed amphetamine and atomoxetine dose-response experiments only. Animals received drug doses according to a fully randomized design, with the experimenter blind to treatment. It is possible that animal’s performance was influenced by the previous drug tested, which could have been mitigated if all drugs and doses had been tested in a fully randomized design. However, this would have increased the number of factors and reduced power and would have required a much larger sample size. Drugs were administered twice per week following a drug cycle of baseline Monday and Thursday, test session Tuesday and Friday, and no treatment or testing on Wednesday or over the weekend. At least 8 days of drug-free baseline sessions were performed before commencing the next treatment, and the animal’s baseline performance was analyzed to check that they were stable before beginning the next drug. A RM ANOVA with TIME as factor was used to assess for effects on baseline performance, but there were no significant effects observed. An acclimatizing dose of nicotine 0.1 mg/kg was administered to all rats 2 days before the start of the dosing regimen. This was only done for nicotine, because previous studies had found animals show a variable response to the first exposure to nicotine. The highest dose of nicotine (0.3 mg/kg) was administered separately after the lower doses were found to be ineffective.

### Performance measures

In this new task, we were able to record a number of different parameters, which we suggest may align to different aspects of attentional processing and impulsive behaviour. For each trial sequence, a single outcome was recorded and any response terminated the trial sequence with a new sequence initiated by the animal following either consumption of the reward (correct trial) or a time-out (incorrect or omission). Correct or incorrect responses recorded during the sequence also generated a response latency. Omissions were recorded when the sequence reached the end, and no response was recorded. Omission can arise either because the animal fails to detect and respond to the target image or because they are not attending to the screen. Because animals must initiate the start of each sequence of images, total omissions in this task were less influenced by overall task engagement, and we can use total trials initiated to make inference about this. When animals make a correct response, latency to collect the reward is also recorded.

Using the data obtained for correct versus incorrect responses, we were able to calculate a number of different variables and, using this range of measures, can make some inference about the different aspects of attention and impulse control, which might influence performance. % Accuracy (correct trials divided by total number of correct and incorrect trials *100) (omitted trials were analyzed separately), % incorrect (incorrect trials divided by total number of accuracy and incorrect trials *100), and % omissions (omitted trials divided by total number of omitted, correct, incorrect trials *100) were calculated for the whole session alongside total trials completed similar to the data report for the 5-CSRTT and 5-CPT. The normalization of the data takes into account the relative proportion of trials completed and, for correct and incorrect responses, that the same motor effort needed (Bari et al., 2008). Data for the individual responses to each image were expressed as % responses for each image (number of responses per image divided by the total number of image responses for correct and incorrect trials*100), where % responses for the target is equivalent to % accuracy, and the sum of the distractor responses is equivalent to % incorrect.

Correct latency and collection latency represent the time taken to respond to the target image and to collect reward respectively, averaged across the total number of accuracy trials. Response latency is the time taken to respond to an image following sequence initiation, averaged across the number of accuracy and incorrect trials. Incorrect latency represents the time taken to respond to a distractor image, averaged across the number of incorrect trials. For cohort 2, the latency to respond to the false-alarm image was calculated separately from the other distractor images (incorrect latency). All latency data are presented in seconds (s) and only those with >0.2 s included in analysis, based on minimum time needed to process and initiate a motor response to touchscreen images in rats (Hirokawa et al., 2011; Reinagel, 2013). Latencies <0.2 s therefore were interpreted as delayed responses to the previous image rather than the current image.

Because of the absence of discrete no-go trials, false-hit, and correct rejection responses, d’ could not be analyzed compared with other rodent CPTs (Mar et al., 2017; Young et al., 2009). However, we were able to plot the data for correct responses relative to the position of the target image in the sequence to obtain attention curves. Attention curves were only calculated where a main effect on accuracy was observed. Data were expressed as % accuracy of responding to the target image in each sequence position (1^st^ to 6^th^ image presented). This analysis specifically looked at responses to target images, and the resulting attention curve could be influenced by either a change in impulse control or impairments in sustained attention. However, by also looking at the results for accuracy by image type, we can see whether the pharmacological treatment causes a change in impulsive responding, which parallel the effects on attention, i.e., are related, or, whether changes in the attention curve arise in the absence of any changes in impulse control. Sustained attention tasks assess the ability of the animal to monitor intermittent and unpredictable events over a sustained period of time (Wicks et al., 2017). By presenting animals with a continuous sequence of random stimuli over a prolonged period but requiring them to monitor and detect the correct stimuli, we suggest that this RSVP task, particularly the attention curves, may provide a measure of sustained attention. Rodent and human psychomotor vigilance tasks (PVTs) require subjects to respond to stimuli randomly presented within a fixed period of time. Decreases in vigilance may be observed by slower reaction times, increases in omissions, and increases in premature responding (Bushnell & Strupp, 2009). As for other rodent attention task, in this RSVP task we are able to extract these same measures, thus providing us with a measure of vigilance. In the final modification to the task where we introduce the false alarm, we can then measure responses to the target versus this near target image against the other distractor images. This has the potential to provide a measure of discriminative or perceptual accuracy.

### Statistical analysis

Data were formatted and performance measures calculated using MATLAB^®^ for Windows (MathWorks Inc version R2015a, USA, https://uk.mathworks.com/). Predrug and dose-response performance measures were analyzed by using separate repeated-measures ANOVA (RM-ANOVA) with session or dose as within-subject (ws) factors. A one-sample *t*-test was used to check performance levels were above chance (17%) during predrug baseline performance. Each drug study was analyzed as an independent experiment. Image responses were analyzed by using separate RM-ANOVA with session and image, or dose and image, for predrug and dose-response data, respectively. Accuracy at each target position in the sequence (1^st^ to 6^th^) was analyzed by using separate RM-ANOVA with session and position, or dose and position, for predrug and dose-response data, respectively. Image responses and accuracy curves were analyzed in instances where significant main effects on attention (% accuracy) were found (amphetamine and atomoxetine only).

Data were checked for normality by using Shapiro-Wilk. Only the data for the attention curve for the high dose of amphetamine in cohort 2 was found to deviate significantly. This was because of one animal, which would meet criteria for an outlier (2 standard deviations from the mean). If this animal was removed, the data were normally distributed. As the majority of the data met the requirements for parametric analysis and 2 factor nonparametric ANOVA is not straight forward, we proceeded with RM-ANOVA analysis. Where significant main effects were observed, Sidak post-hoc tests were used to further analyze the differences between groups. Degrees of freedom were adjusted to more conservative values using the Huynh-Feldt epsilon correction for instances of sphericity violation according to Mauchly’s test, and the data were checked for the assumption of homogeneity of sample variance by using Levene’s test. Epsilon values (ɛ) are stated where the degrees of freedom have been corrected; alpha level was set at 0.05. SPSS for Windows (IBM version 23, USA, https://www.ibm.com/analytics/spss-statistics-software) was used for statistical analysis. Sample size was based on previous studies using similar behavioral tasks. Graphs were plotted using Prism 7 (GraphPad, USA, https://www.ibm.com/analytics/spss-statistics-software).

## Results

### Baseline data

#### Cohort 1

Animals for both cohorts were able to discriminate between the target image and distractors (including the false-alarm image used for cohort 2) and achieved a stable baseline performance (cohort 1: Fig. 2A, Supplementary Table S2; F_2,22_ < 0.71, *p* > 0.501; cohort 2: Fig. 2D, Supplementary Table S2; Session F_2, 22_ < 2.93, *p* > 0.074). Animals could discriminate the target from distractor images above the level of chance (17%) (cohort 1: Fig. 2A; Sessions 1–3 *t*_11_ > 9.20, *p* < 0.001; cohort 2: Fig. 2D; Sessions 1–3 *t*_11_ > 4.68, *p* < 0.001). Target responses were significantly higher than responses to distractor images and the introduction of the false alarm with cohort 2 resulted in a higher level of responding for this image relative to the other distractors (cohort 1: Fig. 2B; Image F_1.2, 13.1_ = 89.36, *p* < 0.001, ε = 0.24, Session F_2, 22_ = 3.48, *p =* 0.049, Image*Session F_6.9, 76.3_ = 0.68, *p =* 0.689, ε = 0.69, spider vs. vase/wheel/heart/flower/dove, *p <* 0.001; cohort 2: Fig. 2E; Image F_1.6,18.1_ = 38.59, *p* < 0.001, spider vs. 4-leg spider *p* = 0.014, 4-leg spider vs. all other distractor images *p* < 0.001). Using an analysis of accuracy over time, we observed that responses declined with lower accuracy when the image was presented in positions 5 or 6 (cohort 1: Fig. 2C; Position F_2.4,26.1_ = 57.17, *p* < 0.001, ε = 0.48, 1 vs. 5–6 *p* < 0.001, 1 vs. 2–4 *p >* 0.05, Session F_2,22_ = 0.71, *p =* 0.500, Session*Position F_10,110_ = 1.23, *p* = 0.278; cohort 2: Fig. 2F; Position F_3.8,41.7_ = 42.86, *p* < 0.001, ε = 0.76, 1 vs. 4–6 *p*<0.01, 1 vs. 2–3 *p* > 0.05, Session F_2,22_ = 1.07, *p* = 0.359, Position*Session F_10,110_ = 0.70, *p* = 0.720). Baseline data also were analysed for each drug study to check if animal’s performance remained stable across the 2-week testing protocol. No significant differences were observed during any of the drug testing protocols (data not shown).

### Amphetamine dose response

Similar findings for amphetamine were observed for both cohort 1 and 2. Analyzing the attention curves revealed higher accuracy when the target image was presented in the first position in the sequence but reduced accuracy for the later images. Cohort 2 did not show any relative difference in response errors for false alarm image versus the other distractor.

#### Cohort 1

**Fig. 3:**
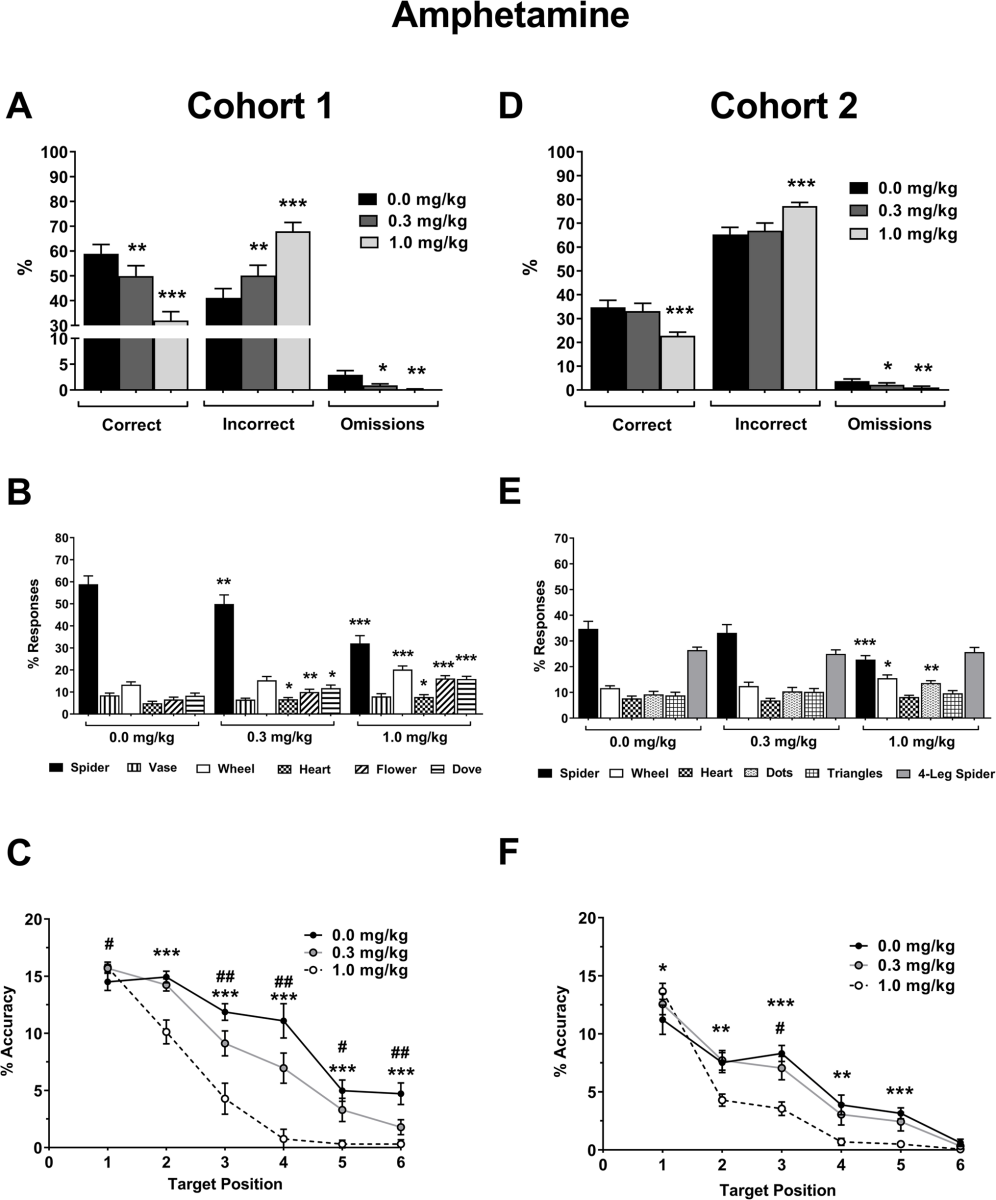
Effect of amphetamine on performance in the rat-rapid serial visual presentation task (R-RSVP). Performance data for cohort 1 (**A–C**) and cohort 2 (**D–F**), response data for % correct, % incorrect, and % omissions for cohort 1 (**A**) and cohort 2 (**D**). Image responses for cohort 1 (**B**) and cohort 2 (**E**), spider is the target image. The sum of distractor responses (all images except spider) is equivalent to incorrect responses for each dose in (**A**). Attention curves showing accuracy per target sequence position for cohort 1 (**C**) and cohort 2 (**F**). Results are shown for the total population, mean ± SEM, n = 12 animals per cohort. Response data (**A, B, D, E**); *p < 0.05, **p < 0.01, ***p < 0.001, versus vehicle (within-subject). Accuracy per target sequence position (**C, F**); #*p* < 0.05, ##*p* < 0.01, 0.3 mg/kg, *p < 0.05, **p < 0.01, ***p < 0.001, 1.0 mg/kg, vs. vehicle (within-subject)

Amphetamine reduced overall accuracy and increased incorrect responding (Fig. 3A; Dose F_2, 22_ = 64.45, *p* < 0.001, vehicle vs. 0.3 mg/kg *p* = 0.002, 1.0 mg/kg *p* < 0.001), but also reduced omissions (F_1.1,12.4_ = 8.62, *p* = 0.010, vehicle vs. 0.3 mg/kg *p* = 0.038, 1.0 mg/kg *p* = 0.006). The number of correct trials declined with dose (Supplementary Table S3; F_2, 22_ = 55.72, *p* < 0.001, vehicle vs. 0.3 mg/kg *p* = 0.009, 1.0 mg/kg *p* < 0.001), but the total number of trials performed by animals was unaffected by treatment (Supplementary Table S3; F_2, 22_ = 1.94, *p* = 0.167). Response latency was reduced at all doses tested (Table 1; Dose F_2,22_ = 57.71, *p* < 0.001, vehicle vs. 0.3 mg/kg, 1.0 mg/kg *p* < 0.001), but other latency measures were unaffected (Table 1; dose F_2, 22_ < 2.86, *p* > 0.079). Amphetamine treatment differentially effected responding depending on the image (Fig. 3B; Image F_1.4, 15.3_ = 65.53, *p* < 0.001, ε = 0.28, Dose F_2, 22_ = 3.92, *p* = 0.035, Image*Dose F_10, 110_ = 39.12, *p* < 0.001, ε = 0.68) with increased responding for all but the vase image, although this was close to significance (*p* > 0.066). Accuracy increased following 0.3-mg/kg dose when the target image was presented at the earliest sequence position (Fig. 3C; Dose F_2,22_ = 64.52, *p* < 0.001, Position F_2.8,31.2_ = 148.83, *p* < 0.001, ε = 0.70, Position*Dose F_7.4,81.8_ = 9.59, *p* < 0.001, ε = 0.74, Position 1 *p* = 0.019) but reduced for later target positions (Positions 3–6 *p* < 0.041). The highest dose reduced accuracy when the target image was presented following target positions 2–6 (*p* < 0.001).

**Table 1:** Latency data for amphetamine and atomoxetine. The effect of amphetamine (AMP) and atomoxetine (ATO) on latency measures in the rat-rapid serial visual presentation task (R-RSVP) for cohort 1 (3-s image presentation) and cohort 2 (2-s image presentation). Only images used with cohort 2 contained a false-alarm image (4-leg spider); therefore, false-alarm latency for cohort 1 was not recorded. Results are shown for the total population, mean ± SEM, n = 12 animals per cohort, measures found to be significantly different compared to vehicle control are indicated in bold and *p < 0.05; **p < 0.01; ***p < 0.001, vs. vehicle (within-subject). *AMP* amphetamine; *ATO* atomoxetine

Cohort	Drug	Dose (mg/kg)	Correct latency (s)	Incorrect latency (s)	False-alarm latency (s)	Response latency (s)	Collection latency (s)
1	AMP	0.0	1.10 ± 0.04	1.46 ± 0.06	-	6.77 ± 0.26	1.56 ± 0.09
		0.3	1.04 ± 0.04	1.50 ± 0.04	*-*	5.60 ± 0.32***	1.47 ± 0.08
		1.0	1.09 ± 0.04	1.54 ± 0.06	-	3.90 ± 0.33***	1.40 ± 0.10
	ATO	0.0	0.97 ± 0.06	1.53 ± 0.05	-	6.13 ± 0.35	1.56 ± 0.07
		0.3	1.04 ± 0.06	1.49 ± 0.05	-	7.18 ± 0.35**	1.61 ± 0.06
		1.0	1.07 ± 0.06	1.56 ± 0.06	-	8.08 ± 0.20***	1.76 ± 0.07**
		3.0	1.24 ± 0.10*	1.59 ± 0.09	-	9.11 ± 0.29***	1.87 ± 0.07**
2	AMP	0.0	0.87 ± 0.05	0.98 ± 0.03	1.02 ± 0.05	3.77 ± 0.22	1.62 ± 0.11
		0.3	0.90 ± 0.05	1.01 ± 0.04	1.00 ± 0.06	3.31 ± 0.25*	1.47 ± 0.06
		1.0	0.83 ± 0.04	1.01 ± 0.03	0.84 ± 0.04***	2.15 ± 0.18***	1.46 ± 0.08
	ATO	0.0	0.92 ± 0.05	1.01 ± 0.04	1.04 ± 0.04	3.98 ± 0.27	1.61 ± 0.12
		0.3	0.97 ± 0.04	0.96 ± 0.04	1.16 ± 0.06	4.92 ± 0.26*	1.85 ± 0.12*
		1.0	0.90 ± 0.05	0.92 ± 0.07	1.14 ± 0.05	4.96 ± 0.30***	1.83 ± 0.09*
		3.0	1.00 ± 0.06	0.92 ± 0.06	1.17 ± 0.11	5.59 ± 0.25**	1.93 ± 0.11*

#### Cohort 2

Amphetamine reduced overall accuracy and increased incorrect responses at the highest dose (Fig 3D; Dose F_2,22_ = 21.52, *p* < 0.001, vehicle vs. 1.0 mg/kg *p* < 0.001, 0.3 mg/kg *p* = 0.465) but reduced omissions at all doses tested (Dose F_2,224_ = 7.36, *p* = 0.004, vehicle vs. 0.3 mg/kg *p* = 0.031, 1.0 mg/kg *p* = 0.006). The number of correct trials also was reduced at the highest dose (Supplementary Table S3; F_2,22_ = 16.41, *p* < 0.001, vehicle vs. 1.0 mg/kg *p* < 0.001, 0.3 mg/kg *p* = 0.970), but the total number of trials performed was not affected by treatment (Supplementary Table S3; F_2,22_ = 2.98, *p* = 0.072). Response latency was reduced by amphetamine treatment (Table 1; Dose F_2,22_ = 53.77, *p* < 0.001, vehicle vs. 0.3 mg/kg *p* = 0.017, 1.0 mg/kg *p* < 0.001) and reduced the false-alarm latency at the highest dose (Table 1; Dose F_2,22_ = 16.83, *p* < 0.001, vehicle vs. 1.0 mg/kg *p* < 0.001, 0.3 mg/kg *p* = 0.562). No other latency measures were affected (Table 1; Dose F_2,22_ < 3.18, *p* > 0.061). Further analysis showed that the highest dose increased responses to some distractor images:wheel and dots (Fig 3E; Image F_1.5,16.2_ = 46.09, *p* < 0.001, ε = 0.29, Dose F_2,22_ = 4.09, *p* = 0.031, Image*Dose F_5.5,60.4_ = 7.40, *p* < 0.001, ε = 0.55, vehicle vs. wheel *p* = 0.017, dots *p* = 0.002). The highest dose increased accuracy when the target was presented first in the sequence (Fig. 3F; Dose F_2,22_ = 21.63, *p* < 0.001, Position F_2.8,31.2_ = 83.65, *p* < 0.001, ε = 0.57, Position*Dose F_10,110_ = 8.31, *p* < 0.001, vehicle vs. 1.0 mg/kg *p* = 0.019). Accuracy was reduced when waiting time for target presentation increased (target positions 2–5, 1.0 mg/kg *p* < 0.002, position 3 0.3 mg/kg *p* = 0.036), with no effect when the target was presented last (0.3 mg/kg, 1.0 mg/kg *p* > 0.113).

### Atomoxetine dose response

Similar results were observed for both cohorts following atomoxetine treatment. Animals were overall more accurate but also made more omissions. The attention curves show that the improvement in accuracy was related to the later time points in the sequence whilst correct responses to the first image was reduced. In cohort 2, a specific improvement in discrimination between the target and false-alarm image was observed.

#### Cohort 1

**Fig. 4:**
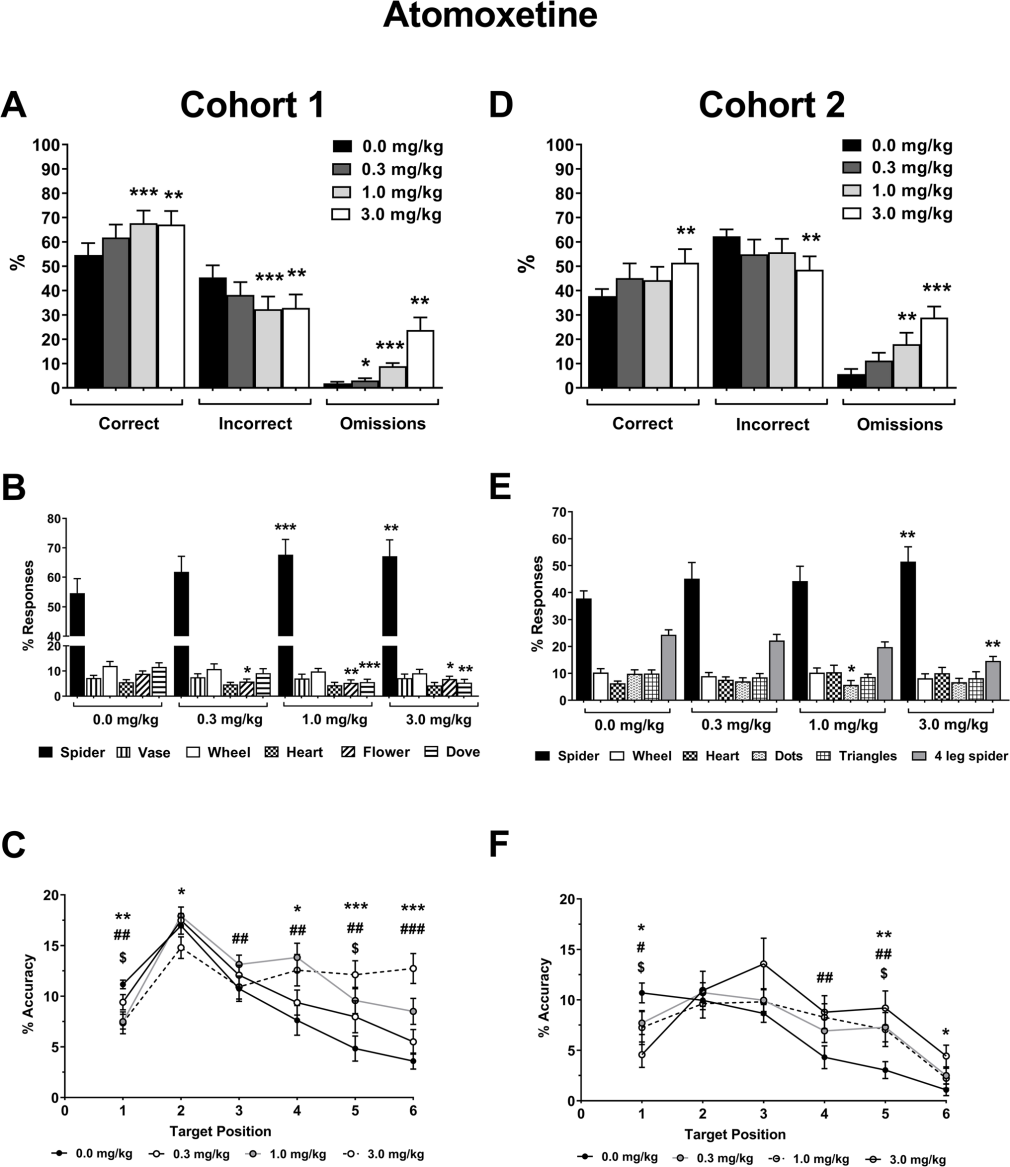
Effect of atomoxetine on performance in the rat-rapid serial visual presentation task (R-RSVP). Performance data for cohort 1 (**A–C**) and cohort 2 (**D–F**), response data for % accuracy, % incorrect, and % omissions for cohort 1 (**A**) and cohort 2 (**D**). Image responses for cohort 1 (**B**) and cohort 2 (**E**), spider is the target image. The sum of the responses to the distractor images (all images except spider) is equivalent to incorrect responses in (**A**). Attention curves showing accuracy per target sequence position for cohort 1 (**C**) and cohort 2 (**F**). Results are shown for the total population, mean ± SEM, n = 12 animals per cohort. Response data (**A, B, D, E**); ^*^p < 0.05, ^**^p < 0.01, ^***^p < 0.001, vs. vehicle (within-subject). Accuracy per target sequence position (**C, F**); ^$^*p* < 0.05, 0.3 mg/kg, ^#^*p* < 0.05, ^##^*p* < 0.01, ^###^*p* < 0.001, 1.0 mg/kg, ^*^p < 0.05, ^**^p < 0.01, ^***^p < 0.001, 3.0 mg/kg, vs. vehicle (within-subject)

Atomoxetine treatment increased overall accuracy and decreased incorrect responses respectively (Fig. 4A; Dose F_3,33_ = 5.19, *p* = 0.005, vehicle vs. 0.3 mg/kg *p* = 0.053, 1.0 mg/kg *p* < 0.001, 3.0 mg/kg *p* = 0.004). Correct trials and total trials were reduced at the highest dose (Supplementary Table S3; F_3,33_ = 6.72, *p* = 0.001, vehicle vs. 3.0 mg/kg *p* = 0.031, and F_1.9,20.5_ = 17.29, *p* < 0.001, ε = 0.62, vehicle vs. 3.0 mg/kg *p* < 0.001 respectively). There was a dose-dependent increase in omissions (Fig. 4A; Dose F_1.3, 13.8_ = 15.49, *p* = 0.001, ε = 0.42, vehicle vs. 0.3 mg/kg *p* = 0.036, 1.0 mg/kg *p* < 0.001, 3.0 mg/kg *p* = 0.001) and response latency (Table 1; Dose F_3,33_ = 36.08, *p* < 0.001, vehicle vs. 0.3 mg/kg *p* = 0.008, 1.0 mg/kg *p* < 0.001, 3.0 mg/kg *p* < 0.004). Collection and correct latency also were increased (Table 1; Dose F_3,33_ = 11.36, *p* < 0.001, vehicle vs. 0.3 mg/kg *p* = 0.320, 1.0 mg/kg *p* = 0.001, 3.0 mg/kg *p* = 0.001, and Dose F_2.0,21.7_ = 6.66, *p* = 0.006, ε = 0.66, vehicle vs. 0.3 mg/kg *p* = 0.135, 1.0 mg/kg *p* = 0.092, 3.0 mg/kg *p* = 0.010, respectively). No effects on incorrect latency were found (Dose F_2.1,22.8_ = 0.41, *p* = 0.675). Analysis of image responses showed that atomoxetine reduced responses to some but not all the distractor images (Fig. 4B; Image F_1.1,12.0_ = 93.16, *p* < 0.001, ε = 0.22, Dose F_3,33_ = 0.50, *p* = 0.682, Image*Dose F_15,165_ = 4.18, *p* < 0.001, flower 0.3 mg/kg, *p* = 0.022, 1.0 mg/kg *p* = 0.001, 3.0 mg/kg *p* = 0.024, dove 1.0 mg/kg *p* < 0.001, 3.0 mg/kg *p* = 0.003).

Accuracy was reduced for the earliest presentation of the target image across all doses (Fig. 4C; Dose F_3,33_ = 5.20, *p* = 0.005, Position F_3.4,36.8_ = 41.34, *p* < 0.001, ε = 0.67, Position*Dose F_15,165_ = 11.23, *p* < 0.001, 0.3 mg/kg *p* = 0.043, 1.0 mg/kg *p* = 0.001, 3.0 mg/kg *p* = 0.004), and for position 2 with the highest dose (*p* = 0.031). However, atomoxetine increased accuracy when the target image was presented in the later target positions 3–6 (Position 3: 1.0 mg/kg *p* = 0.006, Position 4: 1.0 mg/kg *p* = 0.001, 3.0 mg/kg *p* = 0.021, Position 5: 0.3 mg/kg *p* = 0.029, 1.0 mg/kg *p* = 0.001, 3.0 mg/kg *p* < 0.001, Position 6: 1.0 mg/kg *p* < 0.001, 3.0 mg/kg *p* < 0.001).

#### Cohort 2

Atomoxetine treatment increased overall accuracy and reduced incorrect responses at the highest dose (Fig. 4D; Dose F_3,33_ = 5.13, *p* = 0.005, 3.0 mg/kg *p* = 0.005). Omissions increased with atomoxetine treatment (Fig. 4D; Dose F_3,33_ = 11.10, *p* < 0.001, 1.0 mg/kg *p* = 0.009, 3.0 mg/kg *p* < 0.001), as well as response latency and collection latency at all doses tested (Table 1; Response Latency: Dose F_1.8,20.0_ = 7.20, *p* = 0.005, ε = 0.61, 0.3 mg/kg *p* = 0.012, 1.0 mg/kg *p* < 0.001, 3.0 mg/kg *p* = 0.004, Collection Latency: Dose F_3,33_ = 3.37, *p* = 0.030, 0.3 mg/kg *p* = 0.020, 1.0 mg/kg *p* < 0.044, 3.0 mg/kg *p* = 0.024). Atomoxetine also reduced the number of correct and total trials performed (Supplementary Table S3; F_3,33_ = 10.36, *p* < 0.001, vehicle vs. 3.0 mg/kg *p* < 0.001, and F_3,33_ = 35.73, *p* < 0.001, vehicle vs. 1.0 mg/kg *p* = 0.001, 3.0 mg/kg *p* < 0.001 respectively). No other latency measures were affected (Table 1: Dose F_3,33_ < 1.90, *p* > 0.149). Image response analysis showed that atomoxetine reduced responses to distractor images, with the main effect seen for the target versus false alarm, although the lower dose also reduced responses to one of the other distractor images (Fig. 4E; Image F_1.3,14.6_ = 35.95, *p* < 0.001, ε = 0.27, Dose F_3,33_ = 1.77, *p* = 0.172, Image*Dose F_15,165_ = 4.00, *p* < 0.001, 4-leg spider 3.0 mg/kg *p* = 0.002, dots 1.0 mg/kg *p* = 0.026). Atomoxetine reduced accuracy at the earliest target position for all doses versus vehicle treatment (Fig. 4F; Dose F_3,33_ = 5.14, *p* = 0.005, Position F_5,55_ = 21.43, *p* < 0.001, Position*Dose F_15,165_ = 3.20, *p* < 0.001, 0.3 mg/kg *p* = 0.034, 1.0 mg/kg *p* = 0.019, 3.0 mg/kg *p* = 0.019), but increased accuracy at later target positions 4–6 versus vehicle (Position 4: 1.0 mg/kg *p* = 0.004, Position 5: 0.3 mg/kg *p* = 0.012, 1.0 mg/kg *p* = 0.001, 3.0 mg/kg *p* = 0.030, Position 6: 3.0 mg/kg *p* = 0.030).

### Nicotine, Ketamine, Methylphenidate dose response

#### Cohort 1 only

One animal was excluded from the methylphenidate experiment due to a foot injury (n = 11). Nicotine reduced correct latency (Supplementary Table S4; F_4, 44_= 5.73, *p* = 0.001, 0.3 mg/kg *p* = 0.003) and increased collection latency at the highest dose (Supplementary Table S4; F_4, 44_= 4.22, *p* = 0.006, 0.3 mg/kg *p* = 0.043). No other performance variables were affected by nicotine treatment (Supplementary Fig. S1, Supplementary Table S4; F_4,44_ < 1.25, *p* > 0.146). Ketamine increased omissions and reduced the total number of trials performed at the highest dose (Supplementary Fig. S1, Table S4; Dose F_3,33_ = 8.53, *p* < 0.001, 10.0 mg/kg *p* = 0.003 and Dose F_3,33_ = 7.21, *p* = 0.001, 10.0 mg/kg *p* = 0.007, respectively). No other performance variables were affected by ketamine treatment (Dose F_3,33_ < 2.18, *p* > 0.109). Methylphenidate had no effect on any performance variables (Supplementary Fig. S1, Table S4; Dose F_3,33_ < 1.91, *p* > 0.147).

## Discussion

These studies demonstrate that rats are able to learn and perform an RSVP-like attentional task involving a continuous sequence of images. Animals respond to a target image embedded in a randomized sequence of distractors, demonstrating that this method can detect responses to go and no-go targets without having to use discrete trials or interimage intervals. Accuracy levels were overall lower than in previous attentional tasks, such as the 5-CSRTT, potentially making it easier to detect improvements in attention, although it should be noted that only limited success was seen in terms of these pharmacological studies. Analysis of the attentional curves showed accuracy waned over time consistent with reduced ability to sustain attention or withhold responding. By having the concurrent measure of impulsivity across all images, we also can dissociate how drugs influence these two different variables and thus better understand whether the drug is influencing attentional processes or impulse control. The introduction of a false-alarm image gave a clear distinction between target (accuracy), false-alarm, and distractor responses and suggests that it may be possible to distinguish between specific impairments in discrimination or perceptual accuracy (responses to false-alarm image). We did not include a detailed analysis of the impact of different images, and perceptual effects may influence the results, although by using a within-subject design for the drug studies, these are somewhat mitigated. It also may be that with further characterization of different image sets, we may be able to address this limitation and optimize the images and study design. Initial pharmacological investigations suggest that stimulant and nonstimulant treatments have different effects on animal’s performance in this task. Both amphetamine and atomoxetine treatment improved aspects of performance but with very different profiles in terms of the different performance measures recorded. Amphetamine’s effects were limited, and overall accuracy was reduced; however, analysis of the attention curves revealed improvements in accuracy to the target when it was presented early in the sequence, possibly the result of increased vigilance or more impulsive responding or an interaction between both. In contrast, atomoxetine improved animal’s attention curves, suggesting that they were better able to sustain attention during the sequence presentation. Atomoxetine also specifically improved accuracy for the target versus the false alarm.

Training in the task took 42–60 sessions; the most challenging stage for the animals was the introduction of the no-go trials within the sequence. Modifications to the training procedure in the future might further optimize this. The results from the baseline sessions confirmed that rats can distinguish a specific target image within a sequence of distractor images, including a false-alarm image, presented in quick succession. Similar to other attentional tasks, we were able to measure overall accuracy (accuracy vs incorrect), omissions, and both response and collection latencies. In this touchscreen RSVP task, including a false-alarm image for cohort 2 meant that errors of commission (incorrect responses) were more attributable to false-alarm responses than for other nontarget (distractor) images. This enabled the dissociation between effects on discriminative accuracy or perception versus general impairments (responses to all distractor images arising from impulsive responding and/or omitted trials). Under normal conditions, animal’s overall probability of accurately responding to the target image decreased as a function of time, which we suggest could provide a measure of sustained attention. These attentional data, alongside measures of impulsive responding, also may help to differentiate between treatments that increase both impulsive responding and accuracy in rats as suggested by the results with amphetamine. Overall, we are able to measure similar outcomes to other attentional tasks, but our initial studies suggest that this RSVP task may help to dissociate between different aspects of attentional processing. Further investigations are needed, but we suggest that integrating the findings from the different variables and analyses from this task may could potentially dissociate between effects on sustained attention (attentional curve), discriminative or perceptual accuracy (target vs. false alarm), and vigilance (omitted trials, response latency, attentional curves). These are in addition to similar measures of latencies and omissions used to understand effects on motivation and task engagement in the 5CSRTT (Robbins, 2002). A potential advantage of the RSVP task is that the animals cannot predict the presentation of stimuli, thus reducing the influence of procedural learning and timing strategies (Cope et al., 2016).

The level of accuracy shown by both cohorts should be sufficient to detect both improvements and impairments in attention with drugs that are known to alter attentional processing (Cole & Robbins, 1987; Robinson, 2012). This confers advantages over other tasks by allowing the detection of improvements without the need to either change task contingencies or use drug-induced impairments to reduce baseline performance (Mirza & Stolerman, 1998; Young, Meves et al., 2013). However, despite the reduced baseline accuracy seen in these studies is should be noted that, with the exception of atomoxetine, we did not observe improvements in accuracy in our acute pharmacology.

Amphetamine reduced accuracy and omissions in both cohorts and increased the speed of responding to the false-alarm image in cohort 2. Further analysis revealed that a reduction in response time was accompanied by a modest increase in target responses when presented at the earliest time point only. Performance in CPT are sensitive to the effects of stimulant drugs (Servan-Schreiber et al., 1990); amphetamine improved performance and reaction times in normal humans, possibly through increasing vigilance and counteracting fatigue (Rapoport et al., 1980). Translating amphetamine-mediated improvements in performance has been difficult in rodent attention tasks (Baarendse & Vanderschuren, 2012; Cole & Robbins, 1987; Maes et al., 2001), although improvements in attention have been observed in human and mouse 5C-CPTs (MacQueen et al., 2018). In this task amphetamine caused a modest increase in target responses at the earliest time point. However, overall there was no improvement in target versus false-alarm discrimination and responses to all distractor images increased consistent with an increase in impulsive behavior (Baarendse & Vanderschuren, 2012; van Gaalen et al., 2006). Amphetamine is known to increase responding in animals trained in operant tasks (Cole & Robbins, 1987), although increases in dopamine release in areas, such as the nucleus accumbens (Cole & Robbins, 1989; Dalley et al., 2007). An increase in impulsive responding likely contributed to the reduction in accuracy shown by amphetamine in this task. This RVSP task requires animals to sustain their attention for an unpredictable duration, because of the target image being presented at pseudorandom positions in the sequence, which may be useful in understanding the interactions between impulsivity and vigor.

The effects of atomoxetine were very different. Animals were more accurate overall and specifically showed greater ability to discriminate between the target and false-alarm images. They also were better able to sustain their attention with more accurate responses made when the target was presented later in the sequence. However, they also made more omissions and latencies were increased, suggesting there may be some more general effects on task engagement. Across both cohorts, atomoxetine increased accuracy of responding to the target image and improved accuracy in the attention curve analysis. In the second cohort, atomoxetine was found to increase accuracy for the target versus false-alarm image. This improvement in the ability to distinguish between the target and false-alarm image infers that atomoxetine induced specific improvements in attention and the ability to inhibit distractor responding (Navarra et al., 2008). The lack of effect on the other distractor images also suggests that the attention curve effects were not related to a change in impulsive responding in this particular task. The increase in omitted trials is consistent with reported increases in omissions in the 5CSRTT(Baarendse & Vanderschuren, 2012). Response latency was increased, indicating response suppression similar to that observed in a rat CPT using independent images (Mar et al., 2017), although the effects were not consistent. Based on the evidence from the attentional curves, atomoxetine appeared to improve the animal’s ability to wait before responding rather than affecting the ability to speed up recognition of and response to target or nontarget images. No changes in false-alarm latency or collection latency further indicates that animals did not have slowed motor responses in general (Navarra et al., 2008). Taken together, these data suggest that there is a tradeoff between speed and accuracy (Reinagel, 2013), rather than a change in motivation or lack of task engagement (Bari et al., 2008). Animals responded less to the target image when presented in the first or second sequence position but responded more when in the later positions. It is interesting to observe how different these findings are from the touchscreen rCPT results (Ding et al., 2018) where decreased responding was observed across all measures. The effects of atomoxetine on attention observed here may be beneficial in terms of managing some of the symptoms of ADHD, but the findings in this study suggest this may be at the cost of other effects, such as a general slowing of information processing and/or motivation.

In this study, oral doses of methylphenidate had no effect on any of the performance measures. The doses used were similar to those previously reported for the 5-CSRTT and 5C-CPT (Robinson, 2012; Tomlinson et al., 2014). Berridge et al. (2006) has previously suggested that the oral route of administration results in preferential effects on cortical versus subcortical dopamine, potentially providing a more clinically relevant route of administration and less likely to have stimulant-like effects (Berridge et al., 2006). Methylphenidate administered to normal subjects typically reduces errors and reaction times in CPTs (Kollins et al., 2015; Peloquin & Klorman, 1986). Effects in rodent tasks have mainly reported increases in impulsive responding at similar doses to that used here (Milstein et al., 2010; Navarra et al., 2008; Robinson, 2012). The lack of any effects on impulsive responding may relate to the route of administration and/or the format of the task, which reduces the animal’s reliance on timing strategies. Modest improvements in attention in poor performing animals (Navarra et al., 2008; Robinson, 2012) have been reported; however, because of the small number used in this study, analysis based on baseline performance levels was not performed. Differences in the pharmacokinetics of drug administration may explain the lack of effect on attention shown here in animals performing optimally (Berridge et al., 2012; Robinson, 2012). Although most studies in rodent behavioural tasks find similar results for amphetamine and methylphenidate, we have recently observed differences in a naturalistic foraging task in mice with methylphenidate increasing and amphetamine reducing motivation respectively (Xeni et al., 2023). It is therefore possible that the different structure of this attentional RSVP task can differentiate between the effects of these two psychostimulants. It should be noted that this study used animals that had already received other treatments, which may have impacted on the sensitivity to this treatment.

Ketamine had no specific effects on attention in this task but increased the level of omissions at higher doses, suggesting that the animals became disrupted from performing the task. Ketamine’s lack of effect on attention is in-line with our previous study using a modified version of the 5-CSRTT that used an unpredictable stimulus presentation for a more attention demanding task (Benn & Robinson, 2014; Smith et al., 2011). In normal human participants, ketamine reduces accuracy trials and increases omissions and incorrect trials in a CPT (Heekeren et al., 2008; Umbricht et al., 2000). Ketamine-induced errors appear to specifically relate to responses to the target cue (‘X’) and inattention to the cue signal (‘A’), leading to increased responding to ‘B-X’ sequences versus other incorrect combinations that do not contain the target, i.e., ‘B-Y’ or ‘A-Y’ (Heekeren et al., 2008; Umbricht et al., 2000). This suggests that analyzing the type of error is important when assessing ketamine’s effect on performance.

Acute doses of nicotine had no effect on attention but did reduce correct latency in-line with previous reports using similar dose ranges and stimulus durations (Blondel et al., 2000; Grottick & Higgins, 2000; Hahn & Stolerman, 2005). Nicotine-induced improvements in accuracy and response latencies have been reported previously in the 5-CSRTT when task contingencies are changed unexpectedly resulting in impaired baseline performance (Mirza & Stolerman, 1998; Stolerman et al., 2000). However, it is unclear as to how much nonattentional effects (response latencies) contribute to improvements in accuracy in this task. We also found an increase in collection latency at the highest dose, which may reflect effects on the motivation for reward and food-rewarded behaviors (Romero et al., 2018). This is difficult to compare to some key studies (Mirza & Stolerman, 1998; Stolerman et al., 2000) because of a lack of reporting on this parameter.

In drug-naïve humans, the effects of nicotine on attention are inconsistent across different task modalities with effects on commissions (Myers et al., 2013), omissions (Levin et al., 1998; White & Levin, 1999), and reaction times (Bekker et al., 2005; Levin et al., 1996; Myers et al., 2013), all being reported across variants of the CPT. In our task, we found no specific effects on attention or effects on omissions or distractor responses (commission errors). However, nicotine reduced correct latency and preserved target responding, which remains a consistent finding across CPTs (Bekker et al., 2005; Myers et al., 2013; Young, Meves et al., 2013). The lack of effect of nicotine and ketamine also have arisen as a result of animals undergoing multiple drug treatments and repeated testing in the task.

The different profile of effects of atomoxetine and amphetamine highlight possible differences between stimulant and nonstimulant effects on responding that may impact on their clinical benefits in different types of ADHD (Blondeau & Dellu-Hagedorn, 2007; Newcorn et al., 2008). It has been suggested that the effects of these drugs involve similar catecholamine mechanisms within the prefrontal cortex (Berridge et al., 2006; Bymaster et al., 2002); however, our previous studies in noradrenergic lesioned animals suggested differences in their primary sites of action in the brain (Benn & Robinson, 2017). The findings from this rat RSVP task suggest that their effects may involve different mechanisms with amphetamine acting more on maintaining task and cue-elicited responding, whereas atomoxetine improves attention by reducing the speed of responding and improving sustained attention. Further pharmacological studies and experiments involving disease models are needed to help extend knowledge of the validity of this task. These data provide initial support for this task in rats but there are limitations to the study. We only used a limited image data set and did not make a detailed assessment of the perceptual qualities of the different images, and these studies suggest differences that could be better controlled. We also did not undertake the full pharmacological assessment in cohort 2, and further studies are needed to explore the differences between amphetamine and methylphenidate and whether these are specific effects or are related to the dose and route of administration. Only male rats were tested. Further work in females and in mice are necessary before the wider applicability of the task can be established. Finally, the study used a within-subject design, and all animals received multiple drug treatments over the course of the experiment. Carryover effects cannot be fully excluded, although within experiment baseline data suggested the animals’ performance was stable.

### Supplementary Information

Below is the link to the electronic supplementary material.

Supplementary file1 (DOC 398 KB)

Supplementary file2 (MOV 3001 KB)
