# Supplementary material for: Development of a novel rodent rapid serial visual presentation task reveals dissociable effects of stimulant versus nonstimulant treatments on attentional processes
Source: Cogn Affect Behav Neurosci. 2024 Jan 22;24(2):351–67. doi: 10.3758/s13415-023-01152-x (PMC11039523; doi:10.3758/s13415-023-01152-x)
Supplement: Supplementary file 1 — Supplementary file1 (DOC 398 KB) [file 13415_2023_1152_MOESM1_ESM.doc]

**Development of a novel rodent rapid serial visual presentation task reveals dissociable effects of stimulant vs non-stimulant treatments on attentional processes**

Abigail Benn, PhD and Emma S.J. Robinson*, PhD

University of Bristol, School of Physiology, Pharmacology and Neuroscience, Biomedical Sciences Building, University Walk, Bristol, BS8 1TD

*Author for correspondence:

Email: Emma.S.J.Robinson@bristol.ac.uk

Tel: +44 117 331 1449, Fax: +44 117 3312288

**Funding and Disclosure**

Acknowledgements: funding for this research was provided by the Medical Research Council (Ref G0700980 and MR/L011212/1).

**Conflict of interest:** the authors have no conflicts of interest to declare in relation to the work presented in this manuscript. ESJR has current or previous research grant funding through PhD studentships, collaborative grants and contract research from Boehringer Ingelheim, Eli Lilly, MSD, Pfizer and SmallPharma but in areas unrelated to the work presented here.

**Acknowledgements**

The authors would like to thank C. Hales for assistance with analysis using MATLAB®.

**Keywords**

RSVP, attention, amphetamine, atomoxetine, CPT

**Supplementary Methods**

*Training Procedure*

Stage 1 of training involved a conditioned reinforcement schedule (CRF) to reinforce a touch response (nose poke or paw press) to any of the three blank (black) screens. The chamber light was switched off and only the magazine light illuminated for all training and testing. Each screen touch delivered a single reward pellet into the magazine (45 mg Noyes Precision Pellet, Sandown Scientific, UK) to a maximum of 100 pellets. Progression to the next stage occurred if >50 touch responses were made for 2 consecutive sessions.

Stage 2 involved responses to one screen only when a light grey background (LGB) image appeared. The LGB was randomly presented in equal numbers across each screen position (35 trials per screen location), and remained static until touched. Only one touch per trial was allowed, with new trials being initiated automatically. Touches on the blank (black) screens had no effect, only LGB responses were rewarded up to a maximum of 105 rewards (trials). Completion of stage 2 required >50 trials for 2 consecutive sessions. Stage 3 introduced punishment to non-LGB responses by a time-out period in which the house light was illuminated for 10 s. All image parameters the same as stage 2, criterion consisted of >50 trials for 2 consecutive days. For stage 4 animals were required to initiate each trial by nose poking in the magazine on the opposite wall of the chamber once illuminated. All other parameters were kept the same as the previous stage (criteria >50 trials, 2 consecutive days).

Stage 5 marked the start of the image sequence training in which the target image (‘spider’) was presented sequentially with two LGB images. The position of the target image within the sequence (1^st^, 2^nd^, or 3^rd^ position) was randomized and counterbalanced across the total number of trials (120 trials total). Therefore the waiting time for the presentation of the target image varied according to the position in the sequence. Once a trial was initiated each image in the sequence was presented for 3 s, with a total sequence time of 9 s. From this stage onwards, only the centre screen was used, with left/right screens remaining blank (black) and inactive. A response meant a single touch response on the target image and delivered a reward pellet. A single touch on either of the LGB images was classed as an incorrect response. An omitted trial meant that no responses had been made to any image presented during the sequence. Both incorrect and omitted trials were punished with a 10 s time-out and illumination of the house light. Possible trial outcomes, accuracy, incorrect, and omission, are illustrated in figure 1c. Only a single touch response per trial was allowed. To progress to the next stage animals had to perform >60% accuracy (chance performance = 33%), <30% omissions for 2 consecutive training sessions.

Stage 6 of training introduced distractor images into the image sequence. LGB images were replaced by 2 different picture images (Figure 1a, 1b), the total number of images remained at 3. The image sequence (including the distractor images) was randomized across the total number of trials (120 trials) to ensure animals could not predict the occurrence of the target from the presentation of the distractor images. All other parameters remained the same as the previous stage, criteria for progression were >60% accuracy, <20% omissions. For subsequent stages 7-9, additional distractor images were added until the image sequence contained 6 in total (target plus 5 distractors). A blank screen was presented before the image sequence to allow time for the animal to turn round and face the touchscreen once a trial had been initiated. An animal was considered trained once stage 9 was completed (>40% accuracy, <20% omissions, for two consecutive sessions). Chance performance using a 6-image sequence was approximately 17%.

**Additional training for cohort 2 (inclusion of a false alarm)**

The false-alarm image (‘4-leg spider’) was modified from the target image, whereby 4 diagonally opposing legs were removed and a center circle of black pixels replaced by background pixels (figure 1b). Cohort 2 animals also underwent an additional training stage to reduce the image presentation time from 3 s to 2 s to further increase the attentional demands of the task as applied to other rodent attentional tasks. See video 1 for an example of baseline performance in the RSVP task (cohort 2).

***Supplementary Figure S1: The effect of nicotine (a), ketamine (b), and methylphenidate (c), on performance variables % accuracy, % incorrect, % omission, in the rat-rapid serial visual presentation task (R-RSVP).***  *Performance data is for cohort 1 only. Vertical lines indicate that the highest dose of nicotine (0.03 mg/kg) was administered to animals separately to the lower counterbalanced doses (a). Results are shown for the total population, mean ± SEM, n = 12 animals nicotine, ketamine, n = 11 methylphenidate, **p<0.01 versus vehicle (within-subject).*

**Supplementary Table S1: Training stages for rat-ra**pid serial visual presentation task

| Stage | Description | Image Presentation | Criteria |
| --- | --- | --- | --- |
| 1. Touch Training:  Conditioned Reinforcement | Images: 3 x Blank (black)  Screen touch = 1 reward pellet  100 rewards maximum | Continuous until touched | >50 touches  2 consecutive days |
| 2. Touch Training: One Image | Images: 1 x light grey background (LGB), 2 x blank (black)  LGB presented randomly in each screen position (left, right, centre)  LGB touch = 1 reward pellet  105 rewards maximum | Continuous until touched | >50 touches  2 consecutive days |
| 3. Introduction of Punishment | Same as previous stage except responses on blank (black) punished with 10 s time-out | Continuous until touched | >50 touches  2 consecutive days |
| 4. Trial Initiation | Same as previous stage with addition of trial initiation | Continuous until touched | >50 touches  2 consecutive days |
| 5. Sequence Training: Target Image | Images: 1 x target (spider), 2 x LGB  Random image order, presented in centre screen only  Target touch = 1 reward pellet ( trial)  LGB touch = 10 s time-out (in trial)  No touch = 10 s time-out (omitted trial)  120 trials maximum | 3 s / image | >60% accuracy  <30% omissions  2 consecutive days |
| 6. Sequence Training: 3 images | Same as previous stage except LGB replaced with distractor images  Images: 1 x target (spider), 2 x distractor | 3 s / image | >60% accuracy  <20% omission  2 consecutive days |
| 7. Sequence Training: 4 images | Same as previous stage with additional distractor image  Images: 1 x target (spider), 3 x distractor | 3 s / image | >60% accuracy  <20% omission  2 consecutive days |
| 8. Sequence Training: 5 images | Same as previous stage with additional ‘distractor’ image  Images: 1 x target (spider), 4 x distractor | 3 s / image | >50% accuracy  <20% omission  2 consecutive days |
| 9. Sequence Training: 6 images | Same as previous stage with additional ‘distractor’ image  Images: 1 x target (spider), 5 x distractor | 3 s / image | >40% accuracy  <20% omission  2 consecutive days |
| 10. Reduced Presentation Time | Same as previous stage except reduced image presentation time | 2 s / image | >40% accuracy  <20% omission  2 consecutive days |

**Supplementary Table S2: Pre-drug latency** data

| Cohort | Total Trials | Correct  Trials | Correct Latency (s) | Incorrect Latency (s) | False-Alarm Latency (s) | Response Latency (s) | Collection Latency (s) |
| --- | --- | --- | --- | --- | --- | --- | --- |
| 1 | 120 ± 0.1 | 69 ± 5.2 | 1.13 ± 0.05 | 1.45 ± 0.05 | - | 6.72 ± 0.32 | 1.52 ± 0.07 |
|  | 120 ± 0.0 | 69 ± 5.1 | 1.13 ± 0.05 | 1.45 ± 0.05 | - | 6.79 ± 0.30 | 1.50 ± 0.08 |
|  | 120 ± 0.0 | 71 ± 4.6 | 1.10 ± 0.04 | 1.50 ± 0.07 | - | 6.78 ± 0.35 | 1.51 ± 0.07 |
| 2 | 111 ± 2.7 | 37 ± 2.9 | 0.85 ± 0.09 | 0.98 ± 0.04 | 1.01 ± 0.04 | 4.03 ± 0.35 | 1.47 ± 0.07 |
|  | 114 ± 2.5 | 38 ± 3.9 | 0.87 ± 0.04 | 1.00 ± 0.03 | 0.97 ± 0.03 | 3.58 ± 0.27 | 1.46 ± 0.07 |
|  | 116 ± 1.8 | 39 ± 4.7 | 0.89 ± 0.05 | 0.97 ± 0.04 | 1.01 ± 0.03 | 3.75 ± 0.24 | 1.54 ± 0.10 |

Pre-drug latency data for cohort 1 (3 s image presentation) and cohort 2 (2 s image presentation) in the rat-rapid serial visual presentation task (R-RSVP). Only images used with cohort 2 contained a false-alarm image (4-leg spider), therefore false alarm latency for cohort 1 was not recorded. Results are shown for the total population, mean ± SEM, *n* = 12 animals per cohort.

**Supplementary Table S3: Number of total and correct trials** for amphetamine and atomoxetine

| Cohort | Drug | Dose (mg/kg) | Total Trials | Correct  Trials |
| --- | --- | --- | --- | --- |
| 1 | Amphetamine | 0.0 | 119 ± 0.7 | 68 ± 4.4 |
|  |  | 0.3 | 120 ± 0.0 | **59 ± 4.8**** |
|  |  | 1.0 | 120 ± 0.0 | **38 ± 4.2***** |
|  | Atomoxetine | 0.0 | 111 ± 5.4 | 59 ± 6.2 |
|  |  | 0.3 | 116 ± 4.1 | 69 ± 5.9 |
|  |  | 1.0 | 111 ± 3.9 | 67 ± 4.6 |
|  |  | 3.0 | **78 ± 8.4***** | **42 ± 7.7*** |
| 2 | Amphetamine | 0.0 | 111 ± 3.0 | 37 ± 3.2 |
|  |  | 0.3 | 117 ± 2.0 | 37 ± 3.1 |
|  |  | 1.0 | 118 ± 1.6 | **27 ± 1.8***** |
|  | Atomoxetine | 0.0 | 111 ± 5.6 | 40 ± 3.8 |
|  |  | 0.3 | 106 ± 5.2 | 44 ± 7.1 |
|  |  | 1.0 | **80 ± 10.2**** | 32 ± 6.2 |
|  |  | 3.0 | **46 ± 5.8***** | **19 ± 3.9***** |

Total number of trials performed and the number of responses for each drug dose tested in the rat-rapid serial visual presentation task (R-RSVP). Results are shown for cohort 1 (3 s image presentation) and cohort 2 (2 s image presentation), total population mean ± SEM, *n* = 12 animals per cohort, **p*<0.05, ***p*<0.01, ****p*<0.001, versus vehicle (within-subject).

**Supplementary Table S4: Number of trials, responses, and latency data for nicotine, ketamine, and** methylphenidate

| Drug | Dose (mg/kg) | Total Trials | Correct  Trials | Correct Latency  (s) | Incorrect Latency  (s) | Collection Latency  (s) | Response Latency (s) |
| --- | --- | --- | --- | --- | --- | --- | --- |
| Nicotine | 0.0 | 114 ± 3.4 | 63 ± 4.0 | 0.97 ± 0.05 | 1.44 ± 0.05 | 1.64 ± 0.07 | 6.80 ± 0.31 |
|  | 0.01 | 118 ± 1.3 | 62 ± 5.3 | 0.96 ± 0.04 | 1.44 ± 0.06 | 1.57 ± 0.08 | 6.15 ± 0.40 |
|  | 0.03 | 118 ± 1.3 | 66 ± 4.7 | 0.96 ± 0.06 | 1.46 ± 0.03 | 1.64 ± 0.08 | 6.75 ± 0.22 |
|  | 0.1 | 120 ± 0.0 | 70 ± 5.4 | 0.95 ± 0.05 | 1.48 ± 0.04 | 1.59 ± 0.05 | 6.64 ± 0.35 |
|  | 0.3 | 120 ± 0.2 | 68 ± 6.2 | **0.83 ± 0.05**** | 1.50 ± 0.04 | **1.77 ± 0.09*** | 6.40 ± 0.38 |
| Ketamine | 0.0 | 107 ± 5.3 | 59 ± 5.7 | 0.96 ± 0.05 | 1.60 ± 0.05 | 1.72 ± 0.10 | 6.75 ± 0.30 |
|  | 1.0 | 102 ± 7.3 | 55 ± 5.8 | 1.10 ± 0.06 | 1.43 ±0.05 | 1.62 ± 0.09 | 7.09 ± 0.42 |
|  | 3.0 | 105 ± 7.4 | 56 ± 7.0 | 1.10 ± 0.07 | 1.41 ± 0.05 | 1.67 ± 0.08 | 7.19 ± 0.55 |
|  | 10.0 | **77 ± 9.5**** | 45 ± 8.7 | 0.97 ± 0.10 | 1.50 ± 0.09 | 1.66 ± 0.17 | 7.93 ± 0.44 |
| Methylphenidate | 0.0 | 114 ± 4.7 | 62 ± 5.3 | 0.92 ± 0.04 | 1.54 ± 0.04 | 1.60 ± 0.07 | 6.20 ± 0.29 |
|  | 1.0 | 119 ± 1.0 | 59 ± 4.0 | 0.95 ± 0.04 | 1.58 ± 0.05 | 1.50 ± 0.05 | 6.10 ± 0.18 |
|  | 3.0 | 120 ± 0.0 | 62 ± 5.2 | 0.92 ± 0.05 | 1.54 ± 0.04 | 1.54 ± 0.08 | 6.05 ± 0.31 |
|  | 10.0 | 116 ± 2.8 | 55 ± 5.5 | 0.98 ± 0.07 | 1.60 ± 0.05 | 1.56 ± 0.07 | 5.95 ± 0.50 |

The effect of nicotine, ketamine, and methylphenidate on total number of trials, the number of correct trials and latency measures, in the rat-rapid serial visual presentation task (R-RSVP) for cohort 1 (3 s image presentation). Results are shown for the total population, mean ± SEM, *n* = 12 animals nicotine and ketmaine, *n* = 11 methylphenidate **p*<0.05, ***p*<0.01, versus vehicle (within-subject).
